# Excess mortality during the Coronavirus disease pandemic in Korea

**DOI:** 10.1186/s12889-023-16546-2

**Published:** 2023-09-02

**Authors:** Changwoo Han, Hoyeon Jang, Juhwan Oh

**Affiliations:** 1 Department of Preventive Medicine, Chungnam National University College of Medicine, 266, Munhwa-Ro, Jung-Gu, Daejeon, 35015 Korea; 2 Department of Big Data Strategy, National Health Insurance Service, Wonju, Korea; 3 Seoul National University College of Medicine, Seoul, Korea

**Keywords:** COVID-19, Excess mortality, Mortality, Interrupted time series analysis

## Abstract

**Background:**

Although the ongoing epidemics of Coronavirus disease 2019 (COVID-19) may have affected the mortality trend of the nation, the national level assessment of excess mortality (changes in overall mortality in the entire population) is still scarce in Korea. Therefore, this study evaluated the excess mortality during the COVID-19 pandemic in Korea using the certified mortality data.

**Methods:**

Monthly mortality and population data from January 2013 to June 2022 was obtained from the National Health Insurance Service database and Statistics Korea. A quasi-Poisson interrupted time-series model adjusted for age structure, population, seasonality, and long-term trends was used to estimate the counterfactual projections (expected) of mortality during the COVID-19 pandemic (March 2020 to June 2022). The absolute difference (observed—expected) and ratio (observed / expected) of mortality were calculated. Stratified analysis based on pandemic years (years 2020, 2021, and 2022), sex, and age groups (aged 0–4, 5–19, 20–64, and ≥ 65 years) were conducted.

**Results:**

An 8.7% increase in mortality was observed during the COVID-19 pandemic [absolute difference: 61,277 persons; ratio (95% confidence interval (CI)): 1.087 (1.066, 1.107)]. The gap between observed and estimated mortality became wider with continuation of the pandemic [ratio (95% CI), year 2020: 1.021 (1.003, 1.040); year 2021: 1.060 (1.039, 1.080), year 2022: 1.244 (1.219, 1.270)]. Although excess mortality across sex was similar, the adult [aged 20–64, ratio (95% CI): 1.059 (1.043, 1.076)] and elderly [aged 65-, ratio (95% CI): 1.098 (1.062, 1.135)] population showed increased excess mortality during the pandemic.

**Conclusions:**

Despite Korea's successful quarantine policy response, the continued epidemic has led to an excess mortality. The estimated mortality exceeded the number of deaths from COVID-19 infection. Excess mortality should be monitored to estimate the overall impact of the pandemic on a nation.

**Supplementary Information:**

The online version contains supplementary material available at 10.1186/s12889-023-16546-2.

## Backgrounds

Coronavirus disease 2019 (COVID-19) caused by Severe Acute Respiratory Syndrome Coronavirus 2 (SARS-CoV-2) has become a global health issue since the first case was reported in Wuhan, China in December 2019 [1, 2]. As of August 14, 2022, 6.4 million deaths have occurred worldwide due to COVID-19, and 25,623 people have died in Korea [3].

In addition to mortality directly related to COVID-19 infection, changes in overall mortality in the entire population (excess mortality) including COVID-19 death have been reported [4]. Increase in overall mortality were reported in countries with rapid surge of COVID-19 cases, whereas decrease in overall mortality was observed in countries that minimized the epidemic [4–9]. These changes in excess mortality could be attributed to factors such as absolute and/or relative shortage in medical services for non-COVID-19 disease, changes in the incidence of infectious diseases due to increased public hygiene, and changes in healthcare seeking behavior of the public during the pandemic [10–12].

With rigorous contact tracing, testing of all contacts, and implementation of early quarantine strategies, Korea was regarded as one of the countries which successfully managed the COVID-19 pandemic [13, 14]. A previous interrupted time-series study in Korea showed stable mortality patterns during the early pandemic period [10]. No or minimum increase in the mortality of general population were reported in the year 2020 [4, 15].

However, since the third quarter of the year 2021, Korea is showing a rapid surge of COVID-19 patients. Delayed vaccination, emergence of delta variants, and governmental movement for the gradual restoration of normal life may be the causes for the continued increase of COVID-19 cases [16]. Inadequate or overwhelmed capacity to perform testing, contact tracing, isolation, and quarantine during major epidemics may be another reason [17].

With the rapid increase of COVID-19 patients, excess mortality may have been observed in Korea as in other countries. Disrupted emergency medical system and standard pathway for the non-COVID-19 disease management during epidemics [18–21] may have affected the nation’s overall mortality. An increase in response time and poorer prognosis were reported in patients seeking emergency care during the pandemic period in Korea [19–21]. In addition, delays in health screening and non-urgent medical visits were observed during the pandemic [18, 22–26].

On the other hand, non-pharmaceutical measures to manage COVID-19 such as social distancing, regular hand washing, and the use of facemasks may have reduced the spread of infectious disease, and eventually resulted in a decrease in the mortality rate in Korea [8, 27–29]. Decrease in air pollution levels during the pandemic period may contribute to decline in the mortality rate [30].

During the COVID-19 pandemic, above mentioned societal factors affecting the overall mortality of the nation have constantly emerged and changed. However, the national-level assessment of excess mortality during the pandemic is still scarce in Korea. With this perspective, this study aimed to investigate the excess mortality in Korea by investigating if the overall mortality rate of the general population in Korea deviated from the historical trend during the COVID-19 pandemic.

## Methods

### Study design

In addition to daily COVID-19 incidences and death counts, excess mortality compares the observed number of overall mortalities during the pandemic and expected mortalities based on trends during the pre-pandemic period [11]. It is a useful metric to estimate the direct and indirect impact of COVID-19 on the society and enables better comparison between countries with different COVID-19 diagnostic capacities and death registration systems.

The first epidemic of COVID-19 infection in Korea was reported between late February and early March 2020 [13]. The World Health Organization declared COVID-19 as pandemic on March 11, 2020 [31]. Therefore, the starting point of the pandemic in Korea was set as March 2020 as in previous study [10], and the observed and expected mortalities since March 2020 were compared in this study.

### Data inputs

All-cause mortality (date of deaths; sex and age of the dead) data between January 2013 and June 2022 were obtained from National Health Insurance Service (NHIS) database. Information regarding causes of deaths were not opened to researchers due to the validity issue. Overall, information of 2,810,905 (men: 1,523,727, women: 1,287,178) deaths between January 2013 and June 2022 were analyzed in this study.

To adjust for population size of each age group (5-year intervals from 0–4 to 80–84, and 85 years or older) during the study period, the monthly registered population (from January 2013 to June 2022) was obtained from the Korean Statistical Office [32]. To adjust for different age structures across the study period, age-standardized rates were calculated [33, 34].

In addition to the all-cause mortality data from the NHID, the Korea Center for Disease Control (KCDC) provided information regarding COVID-19 patients (sex, age, date of diagnosis, and date of death). However, the information was only available from January 2020 to March 2022 due to the time required for the epidemiological investigation. To estimate the degree of COVID-19 epidemics in Korea, we age-standardized COVID-19 incidence and mortality rates and visually inspected the patterns.

### Statistical analysis

Monthly mortality data from January 2013 to February 2020 and a quasi-Poisson regression model was used to estimate the mortalities during the pandemic period (from March 2020 to June 2022):

$$\mathrm{log}\ {\mu }_{t, x} = {\beta }_{0} + {\upbeta }_{1}{T}_{t} + {\upbeta }_{2}A + {\upbeta }_{3}ns\left(M, df=4\right) +\mathrm{offset }\left(\mathrm{log}\ {P}_{t,\ x}\right) + \varepsilon$$

The numbers of monthly mortality ($\mu_{t,\ x}$: number of mortality on t year-month in *x* age group) were assumed to follow a quasi-Poisson distribution allowing overdispersion. ${T}_{t}$ is the time (in month unit) since January 2013; *A* is the categorical variable representing age groups. *M* is the month indicator variable and 4 degrees of freedom was used to adjust seasonality [10]. $P_{t,\;x}$ is the monthly population in age group *x* in *t* year-month.

The counterfactual (without COVID-19 pandemic) monthly mortality for March 2020 to June 2022 were estimated. Absolute difference (observed—expected) and ratio (observed / expected) of mortality were calculated by comparing estimated and observed mortalities. The 95% confidence intervals (95% CI) for ratio was calculated based on the upper and lower projection values of expected mortalities. Stratified analysis based on pandemic years (2020, 2021, and 2022), sex, and age groups (aged 0–4, 5–19, 20–64, ≥ 65 years) were conducted.

To estimated the overall changes in the mortality patterns during the pandemic, a quasi-Poisson interrupted time-series analysis was conducted by introducing ${\upbeta }_{4}{(T}_{t}-{T}_{0})\cdot { I}_{t}$ term to the regression model. ${T}_{0}$ is the time when pandemic began in Korea (March 2020) and ${I}_{t}$ is the categorial variable representing the time before (${I}_{t}=0,$ from January 2013 to February 2020) and after the pandemic (${I}_{t}=1,$ from March 2020 to June 2022). Based on a previous study showing minimum changes in the mortality rate of the Korean population during the early pandemic [10], a slope change model was selected for the interrupted time series analysis [35]. ${\upbeta }_{4}$ refers to the changes of slope (relative risk) in mortality trend after the pandemic and robust standard errors were calculated [36, 37].

To determine the effects of COVID-19 deaths on excess mortality, we subtract the number of COVID-19 deaths from all-cause deaths and evaluate the monthly age-standardized mortality patterns of non-COVID-19. In addition, monthly COVID-19 and non-COVID-19 mortality rates for each 5-year age groups were evaluated from January 2020 to March 2022. SAS (version 9.4, SAS Institute Inc., Cary, NC, USA) and R statistical software (version 4.0; R Foundation for Statistical Computing, Vienna, Austria) were used. The level of statistical significance was set at a *p*-value < 0.05.

## Results

Figure S1 shows the monthly age-standardized incidence and mortality rates of COVID-19 patients from January 2020 to March 2022 (see Additional file 1). After two waves of the epidemics in the year 2020 (March and December 2020), there has been a continuous increase in the number of COVID-19 patients and mortality since June 2021. The marked increase in COVID-19 incidence and mortality was observed in early 2022.

**Fig. 1:**
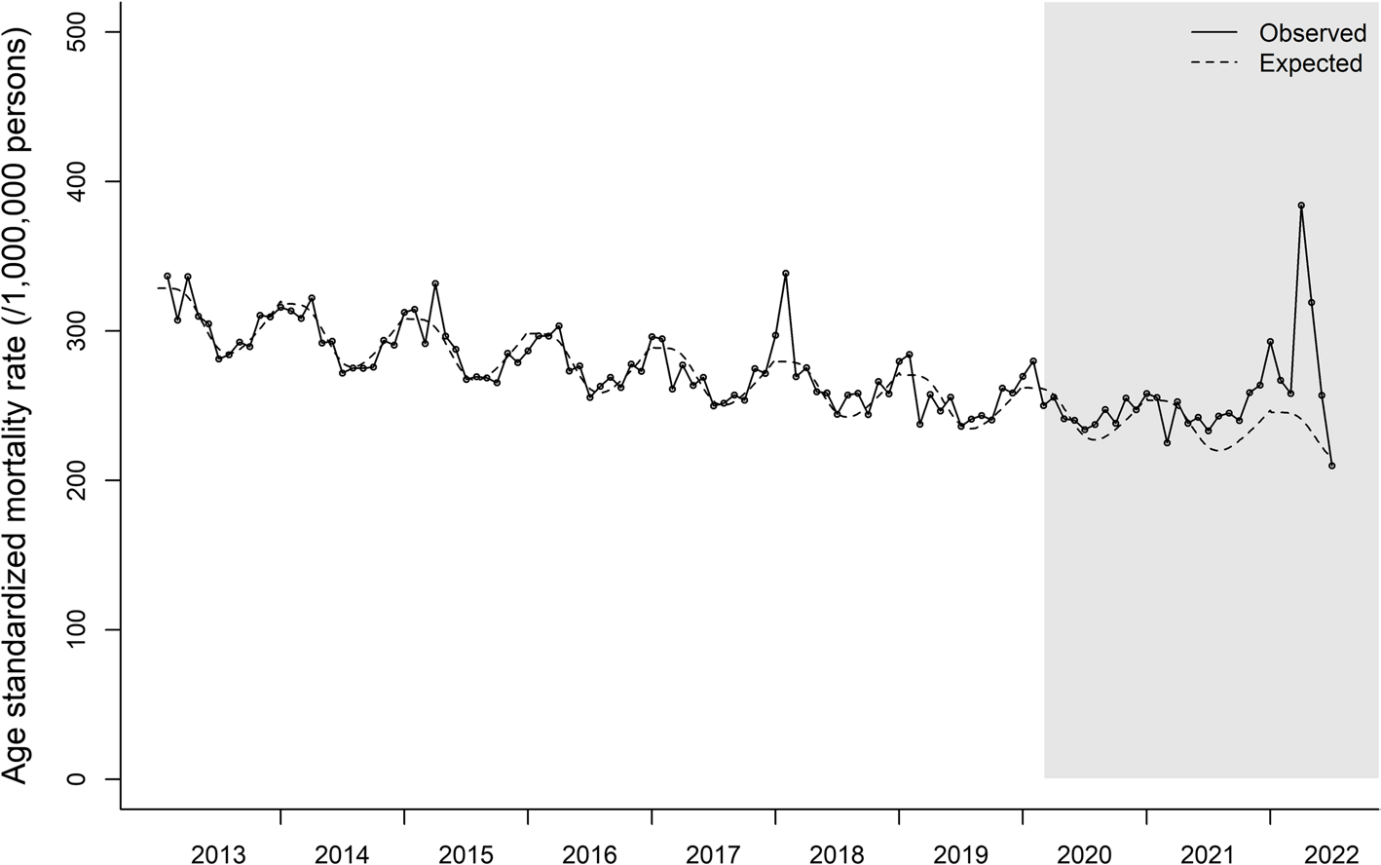
Age standardized mortality rate of Korea from January 2013 to June 2022. Data points and solid line represents age standardized mortality rate. Dotted line represents expected mortality trends estimated based on the pre-COVID19 pandemic (Jan 2013 to Feb 2020) mortality trend

A total of 768,341 all-cause deaths occurred during the COVID-19 pandemic (March 2020 to June 2022) in Korea (Table 1). Deviation from the historical age-standardized mortality trend was observed from the end of 2021 (Fig. 1). The slope change estimate [relative risk (95% confidence interval (CI))] for mortality trend after the pandemic (March 2020) was 1.007 (1.004, 1.010).

**Table 1:** Estimation of excess morality during COVID-19 pandemic in Korea

Period	Observed deaths (N)	Expected^a^ deaths (N)	Difference (N) (observed-expected)	Ratio (95% CI) (observed/expected)	ITS slope change^b^ relative risk (pre vs. pandemic)
Total					
2020.03–2022.06	768,341	707,064	61,277	1.087 (1.066, 1.107)	1.007 (1.004, 1.010)
2020.03–2020.12	254,047	248,771	5,276	1.021 (1.003, 1.040)	
2021.01–2021.12	321,224	303,138	18,086	1.060 (1.039, 1.080)	
2022.01–2022.06	193,070	155,155	37,915	1.244 (1.219, 1.270)	
Men					
2020.03–2022.06	413,080	382,064	31,016	1.081 (1.062, 1.100)	1.006 (1.004, 1.008)
2020.03–2020.12	138,160	134,997	3,163	1.023 (1.006, 1.041)	
2021.01–2021.12	174,067	163,725	10,342	1.063 (1.044, 1.082)	
2022.01–2022.06	100,853	83,342	17,511	1.210 (1.188, 1.233)	
Women					
2020.03–2022.06	355,261	324,795	30,466	1.094 (1.070, 1.118)	1.008 (1.003, 1.012)
2020.03–2020.12	115,887	113,755	2,132	1.019 (0.998, 1.040)	
2021.01–2021.12	147,157	139,291	7,866	1.056 (1.033, 1.080)	
2022.01–2022.06	92,217	71,749	20,468	1.285 (1.256, 1.315)	

^a^Estimated based on pre-pandemic (2013.01 to 2020.02) mortality trend

^b^Interrupted time series (ITS) analysis comparing pre-pandemic and pandemic periods (2020.03–2022.06) slope change. Adjusted for time trend, age structure, and seasonality

Overall 8.7% increase in mortality was estimated during the pandemic [absolute difference between observed – expected number of deaths: 61,277 persons; ratio (95% CI): 1.087 (1.066, 1.107)] (Table 1). The difference between observed and expected mortality became wider with the continuation of the pandemic [ratio (95% CI), year 2020: 1.021 (1.003, 1.040); year 2021: 1.060 (1.039, 1.080), year 2022: 1.244 (1.219, 1.270)].

Observed deaths exceeded the expected deaths during the pandemic among both men [absolute difference: 31,016 persons; ratio (95% CI): 1.081 (1.062, 1.100)] and women [absolute difference: 30,466 persons; ratio (95% CI): 1.094 (1.070, 1.118)]. Men and women both showed increased ratio of death in the year 2022 [ratio (95% CI): men: 1.210 (1.188, 1.233), women: 1.285 (1.256, 1.315)] compared to early pandemic years (years 2020 and 2021).

Table 2 shows the stratified analysis based on age groups. Although there were no differences between the number of observed and expected deaths during the pandemic in young age groups [ratio (95% CI), aged 0 to 4: 0.992 (0.929, 1.059), aged 5 to 19: 1.070 (0.989, 1.157)], observed mortality exceeded the expected mortality in older age groups [ratio (95% CI), aged 20–64 years: 1.059 (1.043, 1.076), aged ≥ 65 years: 1.098 (1.062, 1.135)]. In the year 2022, the estimated difference [ratio (95% CI)] between observed and expected deaths became wider in 0 to 4 [1.096 (1.018, 1.179)], 20 to 64 [1.116 (1.097, 1.135)], and ≥ 65 [1.291 (1.245, 1.339)] years age groups as compared to early pandemic years. However, greatest difference was observed in the year 2021 in 5 to 19 years age group [ratio (95% CI): 1.111 (1.025, 1.203)].

**Table 2:** Estimation of excess morality during the COVID-19 pandemic in Korea (by age groups)

Period	Observed deaths (N)	Expected^a^ deaths (N)	Difference (N) (observed-expected)	Ratio (95% CI) (observed/expected)	ITS slope change^b^ relative risk (pre vs. pandemic)
Aged 0 to 4					
2020.03–2022.06	1,855	1,870	-15	0.992 (0.929, 1.059)	1.001 (0.997, 1.006)
2020.03–2020.12	695	725	-30	0.959 (0.904, 1.018)	
2021.01–2021.12	765	785	-20	0.975 (0.912, 1.043)	
2022.01–2022.06	395	360	35	1.096 (1.018, 1.179)	
Aged 5 to 19					
2020.03–2022.06	4,133	3,864	269	1.070 (0.989, 1.157)	1.005 (1.000, 1.009)
2020.03–2020.12	1,499	1,455	44	1.031 (0.959, 1.107)	
2021.01–2021.12	1,819	1,638	181	1.111 (1.025, 1.203)	
2022.01–2022.06	815	772	43	1.056 (0.967, 1.153)	
Aged 20 to 64					
2020.03–2022.06	203,858	192,488	11,370	1.059 (1.043, 1.076)	1.004 (1.003, 1.006)
2020.03–2020.12	71,176	69,180	1,996	1.029 (1.014, 1.044)	
2021.01–2021.12	87,309	82,648	4,661	1.056 (1.040, 1.073)	
2022.01–2022.06	45,373	40,660	4,713	1.116 (1.097, 1.135)	
Aged 65 or over					
2020.03–2022.06	558,495	508,877	49,618	1.098 (1.062, 1.135)	1.008 (1.004, 1.012)
2020.03–2020.12	180,677	177,316	3,361	1.019 (0.988, 1.051)	
2021.01–2021.12	231,331	218,074	13,257	1.061 (1.026, 1.097)	
2022.01–2022.06	146,487	113,487	33,000	1.291 (1.245, 1.339)	

^a^Estimated based on pre-pandemic (2013.01 to 2020.02) mortality trend

^b^Interrupted time series (ITS) analysis comparing pre-pandemic and pandemic periods (2020.03–2022.06) slope change. Adjusted for time trend, age structure, and seasonality

Figure S2 shows the mortality patterns of all-cause (COVID-19 and non-COVID-19 deaths) and non-COVID-19 mortalities (see Additional file 1). The contribution of excess mortality of COVID-19 deaths increased since December 2021. Figure S3 shows the COVID-19 and non-COVID-19 mortality patterns by each 5-year age groups. There was a marked increase in COVID-19 mortality rates in year 2022 and a larger increase in the older age group (aged over 65).

## Discussion

This study estimated the excess mortality during the COVID-19 pandemic in Korea. Overall, an 8.7% increase in mortality was observed during the pandemic (March 2020 to June 2022). Although the monthly age-standardized mortality of the nation followed the historical trend during the early pandemic, a marked increase was observed since the third quarter of 2021. The increase was greater in the adult and elderly population as compared to adolescents and children.

Underlying mechanism of the excess mortality observed during the pandemic in this study can be speculated as follows. First, the delays and absence of timely and proper medical services since the start of the pandemic could have resulted in late increase in mortality [38]. In a Korean survey with nationally representative sample, 26% and 8% of respondents reported delays in health screening and non-urgent medical visits during the pandemic, respectively [22]. By analyzing the nationwide health insurance claim data which covers all the health facilities of the nation, significant changes in outpatient visits (-9.38%), inpatient admission (-3.82%), hospital visits for diabetes (-2.90%), hypertension (-3.48%), and mental health (-2.60%) were observed in the year 2020 as compared to the previous year [18]. Risk of nosocomial infections and strict implication of social distancing measures may be the reasons for avoiding medical services [10, 18]. Increased mortality can occur months to years after the misses and delays of proper medical services [22, 39].

Second, disruption in emergency medical system may result in immediate increase in mortality rates. Medical resources including emergency departments, ambulances, governmental healthcare officials, and paramedics were mobilized for COVID-19 management in Korea [13, 40]. Therefore, the medical system covering non-COVID-19 emergency patients may have weakened [10]. Since the early stage of the pandemic, the time from the onset of symptom to hospital arrival became longer, and the poor prognosis of the emergency patients were reported [19–21, 41]. Although the in-hospital mortality rate did not change, the out-of-hospital deaths (deaths at home, public facilities, on the way to hospital) increased in Korea in year 2020 [10]. Disruption of emergency medical system may have worsened during the unprecedented increase of COVID-19 patients during the delta and omicron variants epidemics, which began in the third quarter of 2021 (Figure S1).

Third, the capability of critical care management for non-COVID-19 patients may be disrupted. Shortages of intensive care beds, logistics, and trained personnel to manage severe patients were reported in countries with a rapid surge of COVID-19 cases [42–44]. The shortage of hospital beds was also reported in Korea during the first epidemic in early 2020 [45].

Although the Korean government mobilized infectious wards and intensive care beds to prepare for further epidemics, an unprecedented increase of COVID-19 patients since June 2021 (Figure S1) may induce a shortage in the capability for critical care management, which may eventually increase the mortality of emergent COVID-19 and non-COVID-19 disease patients [46, 47].

Fourth, the re-emergence of infectious respiratory disease other than COVID-19 might have attributed to increased mortality during the winter period of 2021–2022. Non-pharmaceutical measures (e.g. hand-washing, facemask use, social distancing) to control COVID-19 were effective in controlling other respiratory diseases worldwide [48, 49]. In 2020, Korea reported a decrease incidence of influenza and respiratory syncytial virus (RSV) infections, as well as overall respiratory mortality [10, 50]. However, the effect of non-pharmaceutical measures to prevent respiratory infectious diseases might have changed due to efforts by the government to gradually restore normal life and decreased participation in social distancing by the public.

For example, unlike the marked decrease in mobility during the early COVID-19 epidemic, there were minimal changes in mobility during the delta and omicron variant epidemics in year 2021 and 2022 in Korea [51]. RSV outbreak reappeared in Korea between November 2021 and January 2022, however, influenza infection remained minimum [51]. The re-emergence of respiratory infectious disease including RSV may have resulted in increased mortality in the winters of years 2021–2022, as observed in this study. However, this must be confirmed with further cause-specific mortality data analysis.

The adult and elderly population showed increased excess mortality during the year 2022. The excess mortality due to COVID-19 deaths increased from December 2021 (Figure S2). Age has been reported to be a significant risk factor for COVID-19 mortality and complications [52]. Therefore, increased infection during the delta and omicron variant epidemics (since June 2021) may have resulted in increased mortality rate in adult and elderly population with COVID-19 infection. The age-group specific COVID-19 mortality pattern in our study also showed a marked increase in the elderly population in year 2022 (Figure S3). In addition, an increased mortality in older age groups from the end of 2021 to 2022 might represent lagged health consequences which appears months or years after the delay or misses of proper medical services [22, 39].

Increase in mortality of ages 5 to 19 during the year 2021 coincides with rapid increase in COVID-19 patients of that particular age group [53]. Although COVID-19 vaccination for adults started in February 2021, the vaccination for adolescents (12 to 17 years) was delayed until October 2021 in Korea [54]. Until the adoption of vaccination, 12 to 17 years age group showed highest incidence since the second quarter of 2021 [53], and this may have resulted in higher excess mortality of that age group during year 2021. Age-specific mortality patterns of the younger age group in our study also showed an increase in COVID-19 mortality since July 2021 (Figure S3).

On the other hand, several studies have reported an increase in suicidal mortalities and a deterioration in mental health in the younger age group in Korea during the pandemic [55, 56]. Although the total number of suicides did not change from the pre-pandemic trends early in the pandemic [10], increasing patterns were seen in women and younger age group (aged ≤ 34 years) [55]. A nationwide cross-sectional survey on Korean adolescents showed increasing patterns of sadness and suicidalities in year 2021 compared to the 15 years of pre-pandemic trends [56]. Unprecedented changes during the pandemic such as school closures, social distancing, and changes in daily activities may have negatively impacted the metal health and its development in children and adolescents [57–59].

Excess mortality can be a useful marker for estimating the impact of COVID-19 pandemic on society [11]. Although absolute number of COVID-19 cases and deaths can be affected by factors like testing capacity, validity of death registration, and political pressure; excess mortality addresses both direct and indirect impacts of COVID-19 [60].

However, mortality represents the end stage health consequences of severe COVID-19 infection cases. COVID-19 patients experience a wide range of health outcomes including long COVID [61]. Therefore, not only the excess morbidity, but also metrics such as disability adjusted life years (DALYs) and quality-adjusted life years (QALYs) should be addressed in the future studies to estimate the true health impact of COVID-19 [38, 61].

The Korean government eased on various policies and measures to prevent COVID-19 infection, such as lifting social distancing and mandatory quarantine of international arrivals, and allowing face-to-face visits for nursing hospitals and facilities since April 2022 [62–64]. This decision was based on high vaccination rate, low fatality rate of COVID-19 patients, and high reservoir of emergency beds [63].

However, Korea has been reporting the highest numbers of new COVID-19 patients in August 2022 [3]. Considering the high excess mortality observed in this study during the time period with marked increase in COVID-19 patients, the mortality rate of recent months would have deviated more severely from the historical trends. Therefore, diseases other than COVID-19 and general medical services for public should be monitored. In addition, eased non-pharmaceutical measures to prevent COVID-19 infection should be reconsidered until the excess mortality became minimum.

This study had some limitations. First, analyses to address which diseases caused the increase in excess mortality were not available due to data constraints. To ensure validity, Statistics Korea is releasing cause-specific mortality data with one year of time lag. Currently, only mortality count data for the years 2021 and 2022 was accessible through the NHIS database. We believed timely analysis for generating scientific evidence was crucial during the pandemic period and conducted this study based on available mortality data.

Second, due to the ecological nature of this study, we were not able to depict particular factors affecting mortality of the Korean. Diverse measures and strategies have been devised and introduced simultaneously in Korea and around the world to combat the pandemic. Since this study is conducted based on single time point distinguishing pandemic from pre-pandemic period (March 2020), the impact of single measures or factors on excess mortality could not be addressed.

Third, the estimation of excess mortality is influenced by several factors, such as the selection of models, definition of baseline and projection periods, and adjustments for potential confounders [4, 65, 66]. In this study, efforts were made to account for long-term mortality trends, and adjustment were made for monthly variations in population age structure and seasonality. The analysis models were selected based on a previous study that evaluated the changes in the overall mortality pattern in Korea during the early stages of the pandemic [10]. However, conducting sensitivity analyses using different analytical choices is crucial to comprehensively assess the mortality burden associated with COVID-19 [65].

## Conclusions

The excess mortality was 8.7% (*n* = 61,277) during the COVID-19 pandemic (between March 2020 and June 2022) in Korea. Excess mortality increased with continuation of the pandemic, showing considerable increase in year 2022. In addition to reporting daily COVID-19 incidence and mortality count, periodic assessment of excess mortality should be conducted to estimate the indirect impact of COVID-19 on the society. Moreover, the government should closely monitor the medical system to manage diseases other than COVID-19 during the pandemic period.

### Supplementary Information

**Additional file 1: Figure S1.** Monthly age-standardized COVID-19 incidence and mortality rates in Korea (from January 2020 to March 2022). Data was provided from the Korea Centers for Disease Control and Prevention Agency (KCDC). The time period used for COVID-19 incidence and mortality data is different from the all-cause mortality data used in the main analysis due to the time that was required for the epidemiological investigation to confirm COVID-19 deaths. **Figure S2.** Age standardized mortality rate of Korea from January 2013 to June 2022. Circled points and the solid line represent all-cause (COVID19 and non-COVID19) mortality rates. Crosshair points represent non-COVID-19 mortality rates (from January 2020 to March 2022). The dotted line represents expected all-cause mortality trends estimated based on the pre-COVID19 (Jan 2013 to Feb 2020) mortality trend. **Figure S3.** COVID-19 and non-COVID-19 death rates (number of deaths/number of population) by 5-year age groups.

## Data Availability

The data that support the findings of this study are available from the National Health Insurance Service, Korea. Restrictions apply to the availability of these data, which were used under license for this study. Data are available from the authors (contact C.H.) with the permission of the National Health Insurance Service, Korea.
