# Supplementary material for: Excess mortality during the Coronavirus disease pandemic in Korea
Source: BMC Public Health. 2023 Sep 2;23:1698. doi: 10.1186/s12889-023-16546-2 (PMC10474701; doi:10.1186/s12889-023-16546-2)
Supplement: Supplementary file 1 — Additional file 1: Figure S1. Monthly age-standardized COVID-19 incidence and mortality rates in Korea (from January 2020 to March 2022). Data was provided from the Korea Centers for Disease Control and Prevention Agency (KCDC). The time period used for COVID-19 incidence and mortality data is different from the all-cause mortality data used in the main analysis due to the time that was required for the epidemiological investigation to confirm COVID-19 deaths. Figure S2. Age standardized mortality rate of Korea from January 2013 to June 2022. Circled points and the solid line represent all-cause (COVID19 and non-COVID19) mortality rates. Crosshair points represent non-COVID-19 mortality rates (from January 2020 to March 2022). The dotted line represents expected all-cause mortality trends estimated based on the pre-COVID19 (Jan 2013 to Feb 2020) mortality trend. Figure S3. COVID-19 and non-COVID-19 death rates (number of deaths/number of population) by 5-year age groups. [file 12889_2023_16546_MOESM1_ESM.docx]

**Additional file 1.**


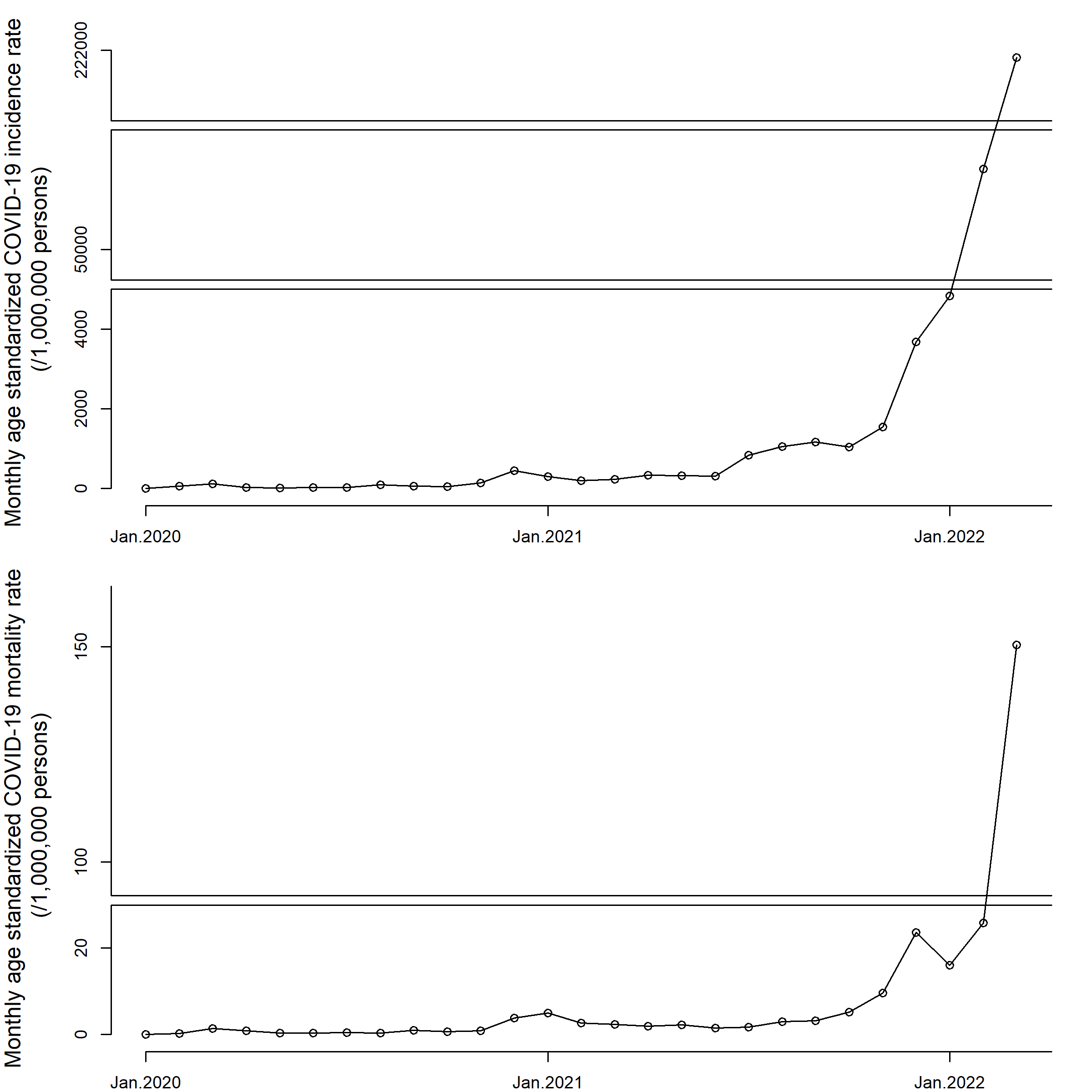


Figure S1. Monthly age-standardized COVID-19 incidence and mortality rates in Korea (from January 2020 to March 2022). Data was provided from the Korea Centers for Disease Control and Prevention Agency (KCDC). The time period used for COVID-19 incidence and mortality data is different from the all-cause mortality data used in the main analysis due to the time that was required for the epidemiological investigation to confirm COVID-19 deaths.


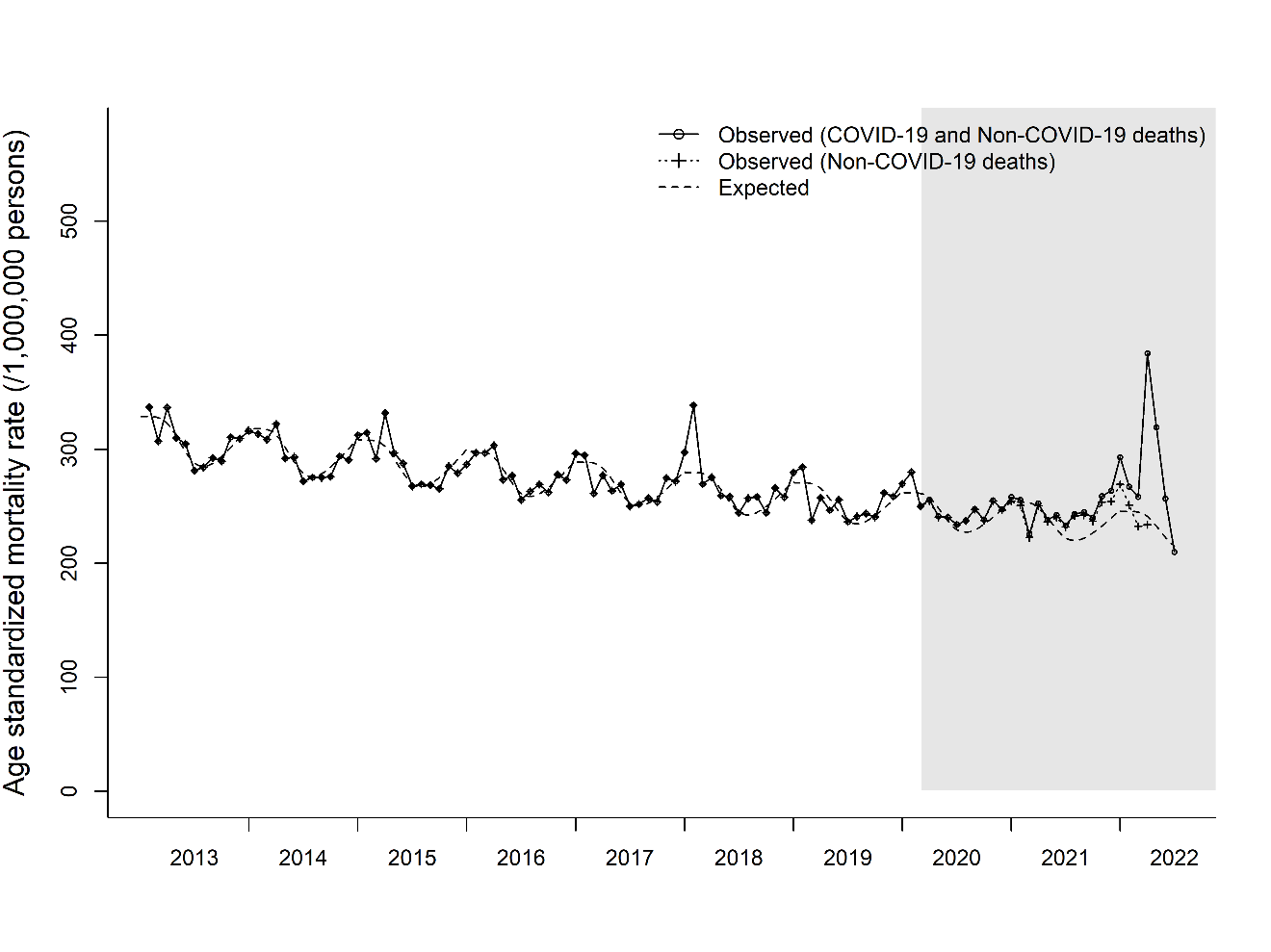


Figure S2. Age standardized mortality rate of Korea from January 2013 to June 2022. Circled points and the solid line represent all-cause (COVID19 and non-COVID19) mortality rates. Crosshair points represent non-COVID-19 mortality rates (from January 2020 to March 2022). The dotted line represents expected all-cause mortality trends estimated based on the pre-COVID19 (Jan 2013 to Feb 2020) mortality trend.


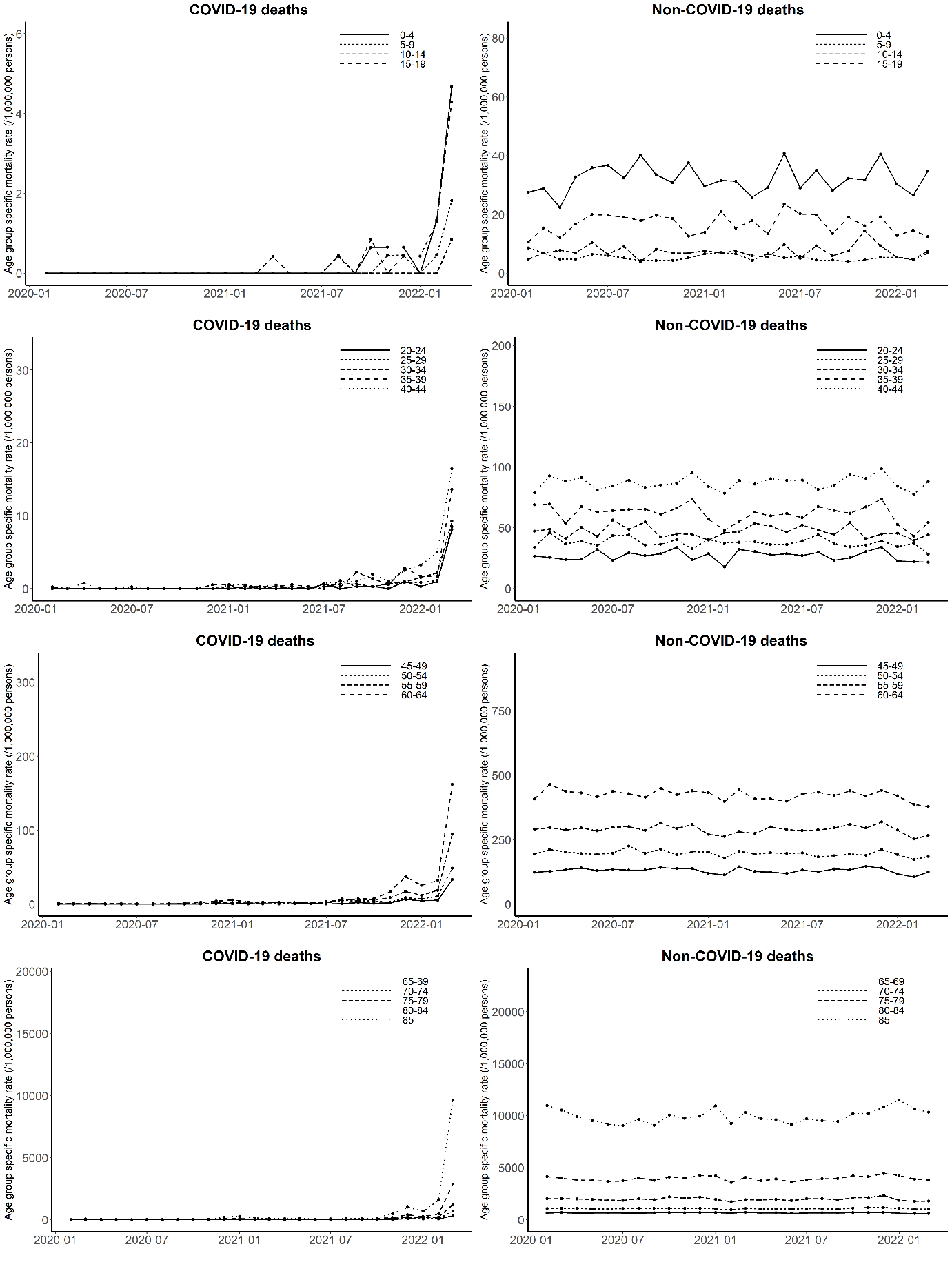


Figure S3. COVID-19 and non-COVID-19 death rates (number of deaths/number of population) by 5-year age groups
